# The Effect of Age on Semen Quality and Spontaneous Pregnancy Rates in Patients who Treated with Microsurgical Inguinal Varicocelectomy

**DOI:** 10.7759/cureus.7744

**Published:** 2020-04-20

**Authors:** Fatih Fırat, Fikret Erdemir

**Affiliations:** 1 Urology, TOKAT State Hospi̇tal, Tokat, TUR; 2 Urology, GOP University Medical Faculty, Tokat, TUR

**Keywords:** varicocelectomy, age, infertility, male, pregnancy

## Abstract

Introduction

The aim of this study is to evaluate whether pregnancy rates or semen parameters are affected due to male or female age after microsurgical varicocelectomy.

Methods

A total of 293 infertile men who underwent microsurgical inguinal varicocelectomy were divided into three groups according to age: group 1, patients and their spouses ≥35 years old (n = 46); group 2, patients ≥35 years old and their spouses <35 years old (n = 34); and group 3, patients and their spouses <35 years old (n = 213). Preoperative and postoperative semen parameters and pregnancy rates were compared.

Results

The median ages of the patients in groups 1, 2, and 3 were 41, 35.50, and 28 years, respectively. The median ages of the spouses were 36 (35-38 years), 30 (21-34 years), and 25 (18-32 years) years, in groups 1, 2, and 3, respectively. Total motile sperm count (TMC) significantly increased in all groups after varicocelectomy (P < 0.05). Pregnancy rates after varicocelectomy was higher in group 3 compared to groups 2 and 3, but the differences were not significant (P = 0.133).

Conclusions

According to these results we can say that male and female ages were not negative factors in terms of pregnancy rates.

## Introduction

Varicocele is an abnormal dilatation of the spermatic veins resulting from insufficient venous drainage and is considered as a common cause of male infertility [1]. Although, the incidence of varicocele in the general male population is approximately 15%, it has been shown responsible in 35-50% of patients with primary infertility and up to 81% of patients with secondary infertility [2]. On the other hand, it is well known that varicocele is a surgically correctable cause of male infertility and numerous studies suggest that varicocelectomy significantly increases sperm concentration, sperm motility and total motile sperm count postoperatively [3].

The traditional method of varicocelectomy is the ligation of the internal spermatic veins under surgical conditions. Several surgical techniques such as retroperitoneal high ligation, radiologic, and laparoscopic approaches have been reported but microsurgery in inguinal or subinguinal varicocelectomy has been performed and quite common. Indeed, microsurgery in varicocelectomy is more effective than conventional techniques regarding pregnancy rates, recurrences and complications [4]. In a meta-analysis of 33 studies, the spontaneous pregnancy rate was 38.37% [5]. Although varicocelectomy is widely used as a treatment option in male infertility, its efficiency has lighted the fuse of a debate in older couples.

The aim of this study is to evaluate the effect of age on microsurgical inguinal varicocelectomy results.

## Materials and methods

Between January 2015 and April 2019, a total of 293 consecutive married, primary infertile men who underwent a microsurgical inguinal varicocelectomy for a clinically palpable varicocele were evaluated retrospectively at our clinic. Patients were divided into three groups according to their ages as follows: group 1, patients and their spouses ≥35 years old (n = 46); group 2, patients ≥35 years old and their spouses <35 years old (n = 34); and group 3, patients and their spouses <35 years old (n = 213). All cases were evaluated with a detailed medical history, complete physical examination, semen analyses, and hormone profile [follicle stimulating hormone (FSH), luteinizing hormone, prolactin, estradiol and total testosterone]. Varicocele was evaluated in the standing position before and during the Valsalva maneuver at room temperature. Patients who could not be examined due to obesity or sensitivity were evaluated with scrotal Doppler ultrasonography. According to the criteria of Dubin and Amelar, a varicocele was classified as grade 1 (palpable only during the Valsalva maneuver), grade 2 (palpable without the Valsalva maneuver), or grade 3 (visible without the need for palpation) [6]. Semen samples were obtained by masturbation after 3-5 days of sexual abstinence and were processed within one hour of ejaculation. A minimum of two specimens were collected, separated by a 2-4-week interval. All analyses were performed in the same andrology laboratory. Preoperative and postoperative total motile sperm counts (TMCs) were calculated using the following formula: TMC = ejaculate volume (mL) × concentration per mL × motile fraction. The spouses of the patients were evaluated for female infertility factors and patients whose spouse might have had concomitant female infertility factors were excluded from the study. Additionally, patients with azoospermia, subclinical varicocele, hormonal pathology (hypo / hypergonadism, hypo / hyperthyroidism, hyperprolactinemia, etc.) that may affect sexual function status, body mass index ≥30 kg/m^2^, using psychiatric drugs, renal, hepatic, cardiovascular and metabolic disease, alcohol and substance addiction were excluded from the study.

All patients with palpable varicoceles underwent a varicocelectomy using a microsurgical artery and lymphatic-sparing surgical technique with an inguinal approach. Postoperative semen analyses were performed every three months for at least nine months after the operation. The average of the two preoperative semen analyses and the last postoperative semen analysis in the first year after surgery were used to compare preoperative and postoperative TMCs.

Statistical analysis

Descriptive analyses related to general characteristics of the study population were performed. The variables were presented as the mean ± standard deviation or n (%). Independent Samples T Test or One-way ANOVA test was used to compare quantitative variables between groups. Also, Chi-Square test was used to analyse qualitative variables between groups. Kruskal-Wallis test was used to compare the same variables among the three groups. Variables are presented as medians and ranges. p < 0.05 was considered as significant. Analyses were performed by using IBM SPSS Statistics for Windows, Version 19.0 (IBM Corp., Armonk, NY).

## Results

The median ages of the patients in groups 1, 2, and 3 were 41 (35-50), 35.50 (35-47), and 28 years (21-33), respectively. The median ages of the patients’ spouses were 36 (range, 35-38 years), 30 (range, 21-34 years), and 25 years (range, 18-32 years), respectively (Table 1). There was no statistically significant difference between the three groups in terms of hormone levels such as FSH, lüteinizing hormone (LH), total testosterone, prolactine and estradiol (p > 0.05) (Table 2).

**Table 1:** Demographic characteristics of the patients Results of Kruskal–Wallis variant analysis between the groups. A statistically significant difference was detected between the groups (P < 0.05). s: spouse; m: men; 35+: thirty-five years old and above; 35-: under thirty-five years.

Groups / n / p	Patient age (years)	Spouse age (years)	Infertility duration (years)
35+ (n = 46)	41.00 (35.00–50.00)	36.00 (35.00–38.00)	7.00 (2.00–16.00)
s35-, m35+ (n = 34)	35.50 (35.00–47.00)	30.00 (21.00–34.00)	4.00 (1.00–8.00)
35- (n = 213)	28.00 (21.00–33.00)	25.00 (21.00–33.00)	2.00 (1.00–9.00)
p	<0.001	<0.001	<0.001

**Table 2:** Distribution of hormonal values by groups A statistically significant difference was detected between the groups (P < 0.05). Data are shown as mean ± SD. s: spouse; m: men; 35+: thirty-five years old and above; 35-: under thirty-five years; FSH: Follicular stimulating hormone; LH: Lüteinizing hormone; T.T.: Total testosterone; Test: One way analysis of variance (ANOVA).

	35+ (n = 46)	s35-, m35+ (n = 34)	35- (n = 213)	p
FSH	2.79 ± 1.19	2.66 ± 1.19	3.81 ± 2.78	0.148
LH	2.88 ± 1.15	3.07 ± 0.74	3.57 ± 1.68	0.168
Prolactin	10.91 ± 4.1	13.15 ± 4.63	10.25 ± 4.08	0.144
T.T.	561.93 ± 193.01	558.93 ± 243.94	505.38 ± 166.55	0.325
Estradiol	24.85 ± 9.22	23 ± 11.53	24.46 ± 8.56	0.880

The differences in semen characteristics were evaluated initially among the three groups before microsurgical ligation. In postoperative period, semen volume significantly decreased in group 1. In the preoperative and postoperative period, it was observed that group 3 was significantly different in terms of semen volume compared to groups 1 and 2 (p < 0.05). Preoperative sperm concentration was detected as 26.61 ± 26.4, 35.43 ± 12.75, and 41.82 ± 38.5 (p = 0.024) in group 1, group 2 and group 3, respectively. In postoperative period these values were detected as 38.14 ± 35.26, 59.21 ± 23.87, and 51.06 ± 50.22 in group 1, group 2 and group 3, respectively. The preoperative and postoperative values ​​between the groups were statistically significant (p < 0.05). The preoperative sperm concentration was significantly different between group 1 and group 3, but there was no statistically significant difference after surgery (p < 0.05).

TMCs increased significantly in all groups after microsurgical varicocelectomy (p < 0.05). Preoperative TMCs were 30.76 ± 42.44, 58.23 ± 35.52, and 54.42 ± 68.58 in groups 1, 2, and 3, and these values increased postoperatively to 57.07 ± 60.22, 167.75 ± 78.6, and 89.04 ± 97.22, respectively. TMC was significantly increased in both groups compared to preoperative period (p < 0.05). After microsurgical inguinal varicocelectomy, the differences in semen characteristics for each group were measured and compared with the preoperative levels (Table 3).

**Table 3:** Distribution of semen parameters before and after varicocelectomy by groups Values marked with different letters (a, b, c) in the same line show those with statistically significant differences. Data are shown as mean ± SD. *A statistically significant difference was detected between the groups (P < 0.05). s: spouse; m: men; 35+: thirty-five years old and above; 35-: under thirty-five years; TMC: total motile sperm count; Pre: preoperative; Post: postoperative; Test: Paired sample T test or One Way Analysis of Variance (#).

		35+ (n = 46)		s35-, m35+ (n = 34)		35- (n = 213)		p#
		n	mean ± SD	n	mean ± SD	n	mean ± SD	
Volume (cc)	Pre	46	3.84 ± 2^(a)^	34	3.82 ± 1.37^(a)^	213	2.89 ± 1.44^(b)^	<0.001*
	Post	46	2.65 ± 1.05^(a)^	34	3.66 ± 1.38^(b)^	213	2.95 ± 1.36^(a)^	0.003*
	P		<0.001*		0.631		0.577	
Concentration (million/mL)	Pre	46	26.61 ± 26.4^(a)^	34	35.43 ± 12.75^(ab)^	213	41.82 ± 38.5^(b)^	0.024*
	Post	46	38.14 ± 35.26	34	59.21 ± 23.87	213	51.06 ± 50.22	0.104
	P		0.037*		<0.001*		0.012*	
Motility (%)	Pre	46	38.14 ± 14.99	34	44.42 ± 12.59	213	42.12 ± 17.97	0.223
	Post	46	48.66 ± 14.79	34	46.39 ± 15.5	213	47.81 ± 16.72	0.826
	P		0.001*		0.551		<0.001*	
TMC (million)	Pre	46	30.76 ± 42.44	34	58.23 ± 35.52	213	54.42 ± 68.58	0.055
	Post	46	57.07 ± 60.22^(a)^	34	167.75 ± 78.6^(b)^	213	89.04 ± 97.22^(c)^	<0.001*
	p		0.004*		<0.001*		<0.001*	

Spontaneous pregnancy rates were 30.4% (n = 14), 41.1% (n = 14), and 46.4% (n = 99) in groups 1, 2, and 3, respectively. Pregnancy rates after varicocelectomy were higher in group 3 compared with those in groups 2 and 1; however, this difference was not statistically significant (p = 0.133) (Table 4).

**Table 4:** Distribution of spontaneous pregnancy by groups Data are shown as n (%). Test: Chi-Square Test *A statistically significant difference was detected between the groups (P < 0.05).

		Spontaneous Pregnancy		p
		-	+	
Groups	35+ (n = 46)	32 (19.3)	14 (11.0)	0.133^*^
	S35-, m35+ (n = 34)	20 (12.0)	14 (11.0)	
	35- (n = 213)	114 (68.7)	99 (78.0)	

## Discussion

Infertility, which is considered a main public health issue, is defined as a couple’s inability to achieve pregnancy following one year of unprotected intercourse. This situation causes loss of self-confidence and social withdrawal in couples. Fifteen to 20% of couples trying to achieve pregnancy are unsuccessful [7]. Male factor contributes in >50% of couples applying to a fertility clinic in primary evaluation. There are many factors such as undescended testis, genetic causes, infections, obstructions of male genital tract, varicocele and environmental factors in the etiology of infertility in men. Although 65% of male infertility is idiopathic, varicocele is another common cause which was diagnosed in 1295 (16.6%) of 7802 men referred for infertility in a European study [8]. Numerous studies have shown the negative effect of varicocele on fertility [9,10].

Varicocele is also the most frequent recoverable cause of male infertility, and previous studies have indicated a significant positive effect of varicocele treatment on semen parameters and pregnancy rates [3]. Although a few types of varicocelectomy methods such as the Palomo technique, Ivanissevich, and subinguinal and inguinal varicocelectomy have been reported, microsurgical inguinal varicocelectomy has gained worldwide application in the treatment of varicocele as it can preserve testicular artery and lymphatic vessels, ligate all spermatic veins, and significantly lower the incidence of complications. Varicocelectomy can improve semen parameters of male infertile patients and increase the pregnancy rates in their female partners. Agarwal et al. metanalyzed 17 studies reporting outcomes of microsurgical varicocelectomy and high ligation varicocelectomy series in infertile male patients [11]. They demonstrated that a microsurgical varicocelectomy significantly improves seminal parameters in infertile men with a palpable varicocele and abnormal semen analyses [11]. Similar results have been reported in several studies [12,13]. In the present study, sperm concentrations increased significantly after a varicocelectomy in groups 1, 2 and 3.

Spontaneous pregnancy rates after varicocelectomy range from 16% to 60%. Watanabe et al. reported a spontaneous pregnancy rate of 50.9% following subinguinal microsurgical varicocelectomy. On the other hand, this rate was 40.4% following laparoscopic approach and 35.8% with the Palomo technique [14]. In a meta-analysis, overall spontaneous pregnancy rates were 37.69% for the Palomo technique, 41.97% for microsurgical varicocelectomy techniques, 30.07% for laparoscopic varicocelectomy techniques, 33.2% for radiologic embolization, and 36% for a macroscopic inguinal varicocelectomy series [14]. Thus, the results of microsurgical varicoceletomy are better than those of other techniques. In our study, the overall pregnancy rate was 46.4%, which was similar to the results of other studies [15,16].

Several parameters such as preoperative sperm count (>5 × 10^6^/ml), FSH level, varicocele grade, testicular size, and Johnsen score predict the success rate of a varicocelectomy [17,18]. In addition, in a limited number of studies it has been reported that male or female age can also be important. Studies, obviously, show an age-related deficiency in female fertility, which can be attributable to many potential causes such as decline in oocyte quality, the frequency and efficiency of ovulation, sexual function, and uterine health [19]. The fertility potential is reduced to 50% at age 35, 25% at age 38, and <5% at >40 years, compared to a 25-year-old woman [20]. Although advanced maternal age may be an important factor in couples’ fertility, the true effect of male or female age on pregnancy after varicocelectomy has not been studied extensively. In 1998, Nieschlag et al. evaluated the pregnancy rates of couples with clinical varicocele in a prospective, randomized study in which varicocelectomy was compared to counseling. The authors revealed that although sperm concentrations had increased significantly only in the surgical group, pregnancy rates were not significantly higher in the surgical group compared with those in the counseling group. They showed that female age at the time of admission was the only parameter associated with positive pregnancy outcome. However, in that study, mean female age was <35 years. The results of our study revealed that female age was not a significant factor for pregnancy rates after a varicoceletomy when we compared all groups [21]. In another study, O’Brien et al. compared the results of two cohorts of infertile men to investigate the effect of female age on pregnancy outcomes following varicocelectomy [22]. Both groups had varicoceles and female partners ≥35 years. One group accepted microsurgical varicocelectomy while the other group chose observation. Sperm count and total motility increased significantly after varicocele repair. Furthermore, 35% of couples in the surgical group achieved spontaneous pregnancy by an average of 30 months after the surgery, whilst only 25% of couples achieved this in the nonsurgical group. These results suggested that surgical varicocelectomy was a viable option even for couples with older females. Our result is similar with those mentioned above; the microsurgical varicocelectomy changes semen parameters, however, pregnancy rates are not significantly different in groups.

Like female age, male age might be an important factor for fertility. Semen parameters decrease with age [23]. In a large retrospective study, a paternal age of ≥40 years was found to be related with a higher risk of delayed pregnancy onset and difficulties conceiving in couples with women aged 35-39 years [24]. Generally, the literature suggests a gradual decline in male fertility with advancing age which becomes prominent especially at the fifth decade and paternal age contributes relatively smaller to the overall age-related decline in the fertility of a couple when compared with maternal age. In another study, patients were subdivided into those <30 years and those ≥30 years to determine the effect of age on varicocelectomy results. The authors indicated that patient age at the time of the operation was of greater value for predicting the treatment outcome of a varicocelectomy [25]. In contrast to that report, the reproductive outcomes of infertile couples with a clinical varicocele and advanced paternal age were evaluated by Zini et al. [26]. The authors included 115 men aged ≥40 years and 466 men <40 years with a clinical varicocele who were infertile. No significant differences were observed in baseline semen parameters or in spontaneous pregnancy rates after varicocelectomy in couples with advanced paternal age compared with those in younger couples (49% vs. 39%, respectively). So, the authors suggested that paternal age did not affect pregnancy outcomes after varicocelectomy negatively. In another study, Ishikawa and Fujisawa reported that male age was not a significant factor for predicting pregnancy outcomes after varicocelectomy. However, the study was small in subject count and was uncontrolled [27]. Tinga et al. reported a recovery in semen parameters only in patients <30 years of age, whereas no significant improvement was observed in older patients. That study was not randomized, double-blinded, or controlled [28]. In another study, 70 male infertile patients with azoospermia or oligospermia who underwent microsurgical inguinal varicocelectomy were evaluated to assess semen characteristics, hormone levels, and pathological findings before and after venous ligation in order to understand whether there is an effect of aging on those characteristics in patients with varicocele [29]. The mean age of the patients was 33.9 ± 5.5 years. Patients were divided into three groups according to the age: group 1, 20-29 years old (n = 17); group 2, 30-39 years old (n = 41); and group 3, >40 years old (n = 12). The results showed that age was not a significant predictor of improved semen characteristics before ligation [29]. Performing a ligation is reasonable to gain an improvement in semen characteristics of patients >40 years old. Similar results have been reported by Resorlu et al. [30]. Although postoperative TMC rates increased significantly in all groups in our study, the pregnancy rates were not significantly different between the groups.

## Conclusions

There is limited information about the effect of varicocelectomy operation on semen parameters in older men. According to the results of this study, it can be concluded that varicocelectomy operation provides acceptable success rates in terms of pregnancy compared to young couples. Similarly, after varicocelectomy operation in older men, it is seen that there is a significant increase in sperm parameters as in young men. Although assisted reproductive methods are traditionally considered in the first step to have children, especially in older couples, varicocelectomy operation in these cases should be performed without waiting for a long time, as it correlates with relatively high pregnancy rates. However, randomized studies in larger population should be performed to confirm these results.
